# Elbow Arthroscopy: Review of the Literature and Case Reports

**DOI:** 10.1155/2012/478214

**Published:** 2012-10-24

**Authors:** Prakash Khanchandani

**Affiliations:** Department of Orthopaedics, Sri Sathya Sai Institute of Higher Medical Sciences, Prasanthigram, Puttaparthi, Andhra Pradesh, India

## Abstract

Elbow arthroscopy, though described first in 1930s, gained popularity only in the last 3 decades. There has been a steady expansion in the clinical applications of elbow arthroscopy owing to the significant improvements in instrumentation and arthroscopic skills. The procedure which was mainly used for diagnostic purpose, loose body removals, and synovial biopsy has now become an important tool for managing elbow arthritis, stiff elbow, and trauma. However, this procedure has a higher incidence of neurological complications and hence case selection and surgeon's expertise are of utmost importance.

## 1. Introduction

Elbow arthroscopy has gained popularity steadily over the last three decades. Though elbow arthroscopy still remains a relatively uncommon procedure to a general orthopaedist; current advanced equipment, increasing experience, and newer techniques have made it a safe and effective tool for diagnosis and treatment of elbow problems [1]. Arthroscopic expertise and anatomical precision are mandatory to establish a safe and reproducible procedure [1, 2].

Till about a decade back elbow arthroscopy was used mainly for loose body removals and diagnostic arthroscopy of the painful elbow [2]. However, present decade has seen significant expansion in the indications of elbow arthroscopy ranging from traumatic elbow pathologies to the arthritic elbow [3].

## 2. Case Reports

**Case 1**

A 40 year old male, carpenter by profession, presented to us with pain in left elbow since 2 years following a fall. The pain was sharp to dull aching and localized mainly to the posterolateral aspect of elbow. To start with, the pain was not significant; however it had deteriorated since last 2 months and was aggravated during heavy works, thus affecting his activity of daily living significantly. On clinical examination, range of motion of elbow was full but painful after 110 degrees of flexion and during pronation/supination. There was no localized swelling and local rise of temperature. Local tenderness was noted on the posterolateral aspect of elbow especially over the anconeus triangle. At the time of presentation, visual analogue scale score for pain was 10 during heavy activities and 7 during light work. There was no neurovascular deficit. Plain radiographs and blood investigations were normal.

**Figure 1:**
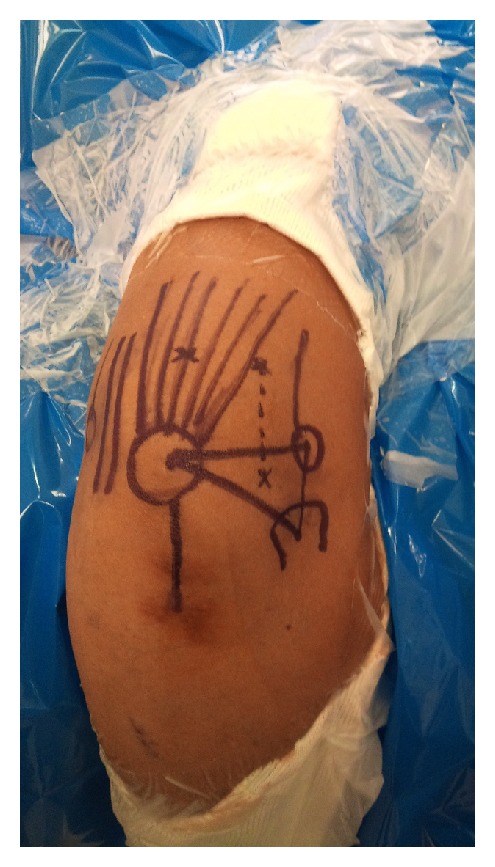
Clinical photograph showing portals for elbow arthroscopy.

Diagnostic elbow arthroscopy was planned. With patient in lateral decubitus position and elbow hanging on the arm holder, elbow arthroscopy was done with a 4 mm 30 degree arthroscope. By using direct lateral portal (Figure 1) in anconeus triangle as the viewing portal, radiocapitellar joint, radial head, proximal radioulnar joint, and coronoid process were visualized. posterolateral and accessory posterolateral portal and direct posterior portals were used to assess the posterior joint and olecranon fossa.

**Figure 2:**
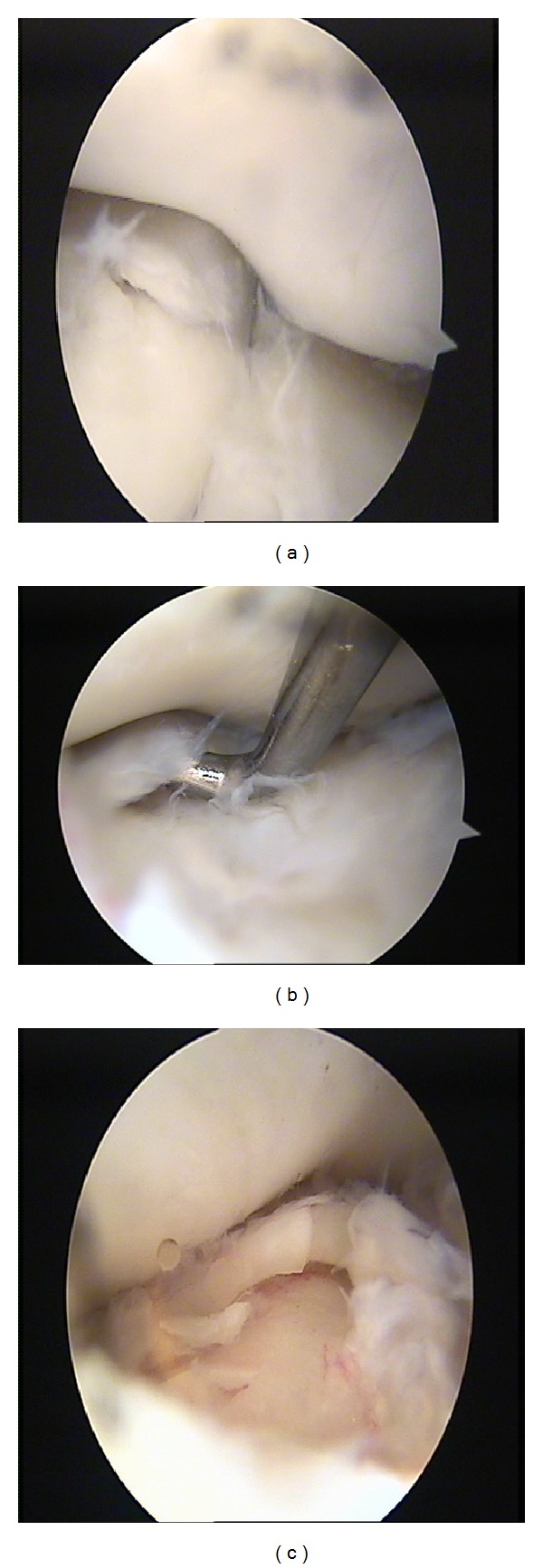
(a)-(b) Photographs showing grade 3 chondral defect on the articular surface of radial head. (c) Photograph showing microfracture following the debridement of the loose cartilage flap.

Radial head was found to have a well-defined chondral defect ICRS grade 3 (Figure 2(a)). The proximal radioulnar joint and ulnohumeral joint were normal. Humeral articular surface was also normal. The chondral defect was debrided and microfracture; abrasion chondroplasty with thermal chondroplasty was done (Figure 2(b)). Patient was put on active rehabilitation schedule from the first postoperative day with all the elbow range of motion exercises. However, lifting weights and heavy work were restricted for a period of 8 weeks.

**Figure 3:**
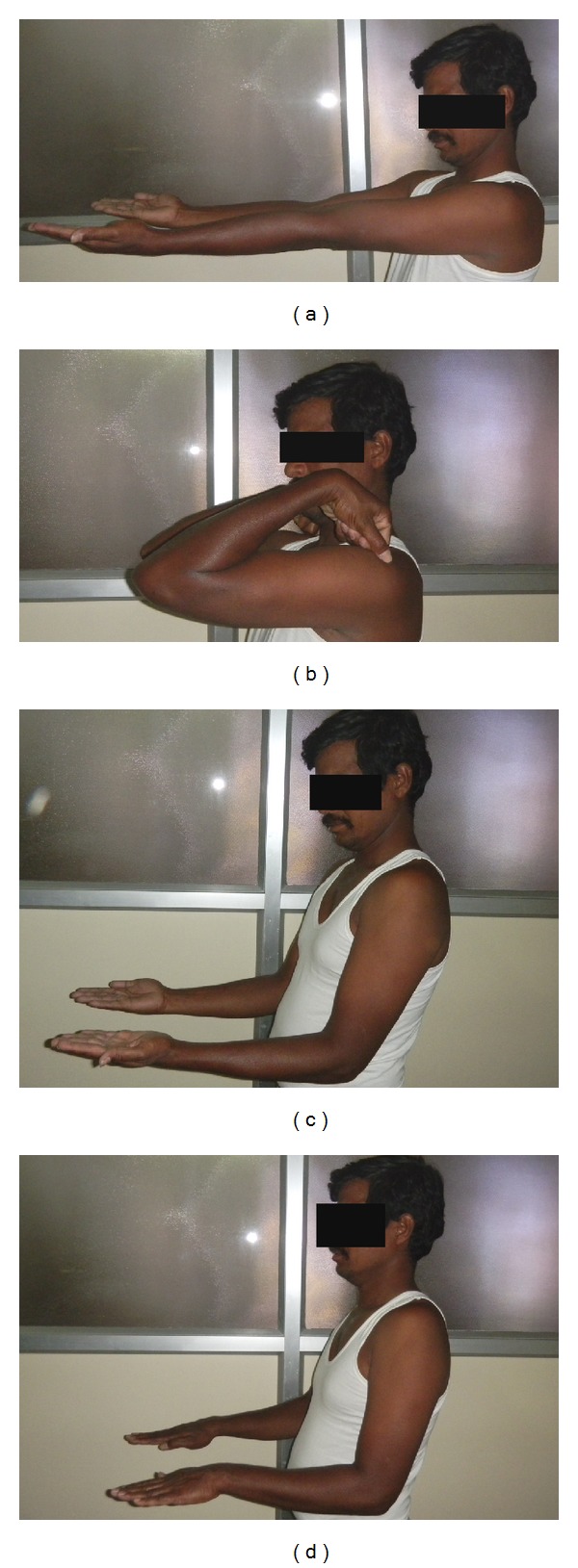
(a)–(d) Clinical photographs of the patient showing ROM at the final followup.

Patient was followed up at 2 weeks, 6 weeks, and 12 weeks and then at 6, 12, and 24 months. Patient regained full painless range of motion of the elbow at the end of 6 weeks, which was maintained at the final followup (Figures 3(a)–3(d)). Patient was permitted to do heavy activities including lifting weights after 12 weeks. At the final followup, patient's pain score on visual analogue scale was 0 during light activities and 1 during heavy activities.

**Case 2**

27 year male presented with a post-traumatic synovitis of left elbow. The patient had sustained a trivial injury to the left elbow 1 year back and he developed a painful swollen elbow which gradually progressed over a period of time. Patient had taken treatment from an orthopaedist which included physiotherapy and anti-inflammatory medications; however he had deteriorated progressively. At the time of presentation, patient had a fixed flexion deformity of 80 degrees with a painful range of motion being 80 to110 degrees. Supination and pronation were also severely restricted and painful with a range of 20 degrees each. The elbow was swollen and extremely tender to palpation especially on the posterolateral aspect. The pain score on visual analogue scale was 10 during routine activities. There was no neurovascular deficit. Radiographs and blood investigations were normal and MRI of elbow revealed generalized synovitis of elbow.

**Figure 4:**
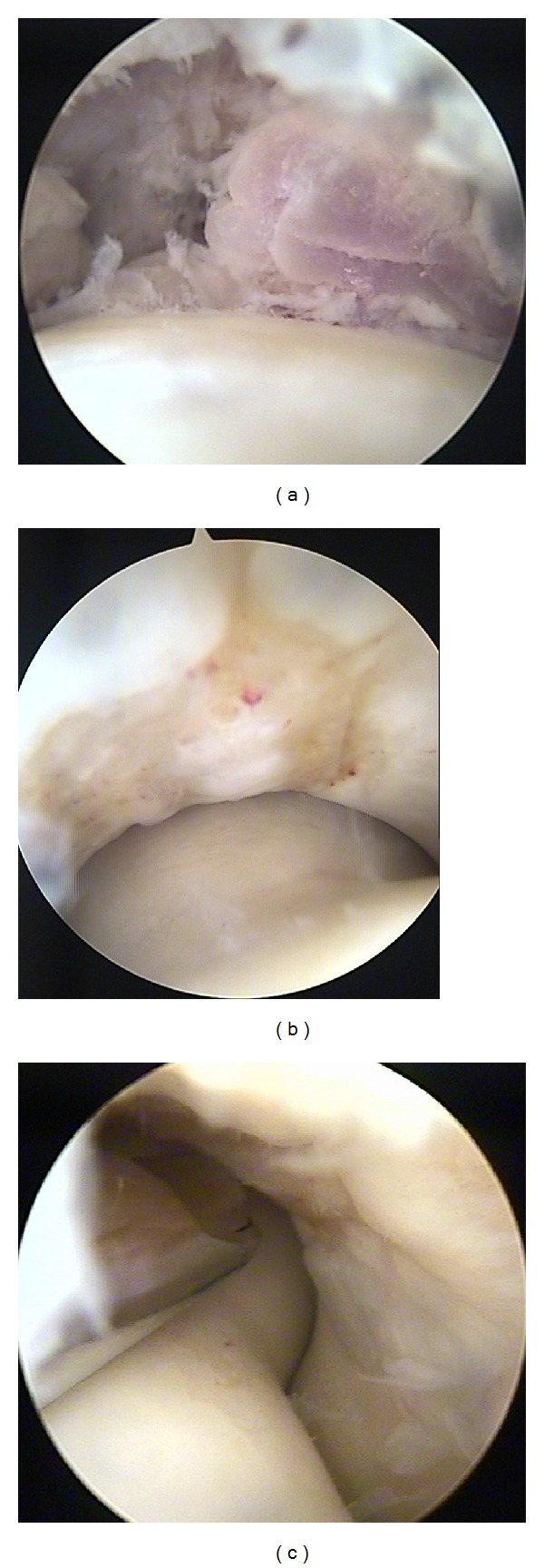
(a) Synovitis in the radiocapitellar joint. (b) Grade 2 chondral lesion on the trochlea. (c) Debridement and abrasion chondroplasty of the lesion with a shaver.

Diagnostic arthroscopy of elbow was done using direct lateral, posterolateral, accessory posterolateral, and posterior portals. There was diffuse synovitis of elbow (Figure 4(a)). A well-defined chondral lesion ICRS grade 2 over the trochlea was detected (Figure 4(b)). The defect was debrided and abrasion chondroplasty (Figure 4(c)) with subtotal synovectomy was done. A compression bandage was given. Patient was started on rehabilitation from the first postoperative day and rehabilitation continued on outpatient basis. Patient was followed up at 2, 6, and 12 weeks and then at 6, 12, and 18 months. 

**Figure 5:**
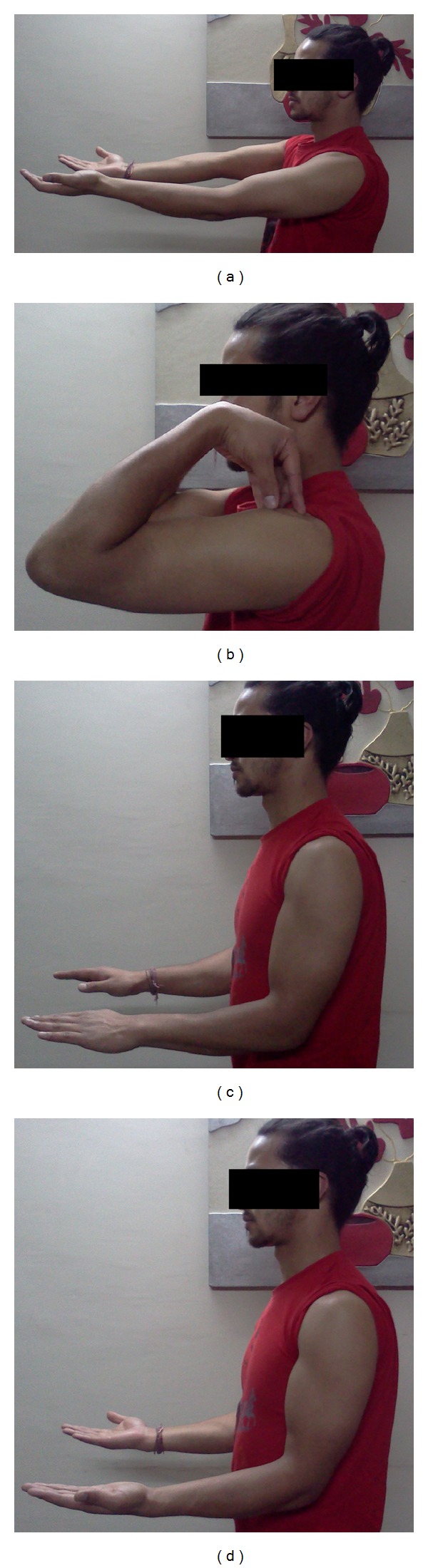
(a)–(d) Clinical photographs of the patient showing ROM at final followup.

Patient gained 30–120 degrees of range of motion of elbow with minimal pain on first postoperative day and the range of motion gradually became painless over a period of 6 weeks. At the final followup after 18 months of surgery, the patient had painless range of motion from 10 to 130 degrees and painless full supination and pronation of elbow (Figures 5(a)–5(d)). Patient was pain-free during all his activities.

## 3. Discussion

Dr. Burman is considered the father of elbow arthroscopy as he tried it for the first time in 1931. In his first attempt he termed elbow joint not suitable for arthroscopy; however he included the elbow joint in the list of joints amenable to arthroscopy a year later [4]. After a huge unexplained gap of about 40 years 1970s and 80s saw a surge in cadaveric studies and exploration of the detailed arthroscopic anatomy of elbow by enthusiastic arthroscopic surgeons like Andrews and Carson [5], Guhl [6], Ward and Anderson [7], and O'Driscoll and Morrey [8]. 

Andrews and Carson [5] published a preliminary report with results of elbow arthroscopy in 12 patients. They documented best results with loose body removals. Ward and Anderson [7] reported their results of elbow arthroscopy in 37 patients in 1992 and they also reported good results with loose body removals and spur excision. O'Driscoll and Morrey [8] evaluated 71 elbow arthroscopies with a mean followup of 37 months. 73% of their patients had benefitted clinically. 

In a retrospective study of 103 elbows, Jerosch et al. [9] noted significant improvement in pain scores and function in degenerative arthritis group. Nemoto et al. [10], Lee and Morrey [11], and Tanaka et al. [12] concluded from their respective studies that elbow arthroscopy is beneficial for rheumatoid elbow. Cohen et al. [13] in a prospective study compared open and arthroscopic elbow debridement and concluded that the arthroscopic group had a better pain relief, while the open group had better ROM. McLaughlin et al. [14] retrospectively evaluated radiocapitellar arthritis treated by arthroscopic radial head excision and reported good results. Peart et al. [15], Rubenthaler et al. [16], and Baker Jr. and Baker III [17] in their studies evaluating results of arthroscopic ECRB release for lateral epicondylitis concluded that it gave a long lasting relief. Takahara et al. [18] in a retrospective series reported good result after arthroscopic management of OCD of capitellum. Menth-Chiari et al. [19] reported good results in 12 patients treated by arthroscopic complete radial head excision. Rolla et al. [20] reported good results after arthroscopic treatment of radial head fractures. Yeoh et al. [21] in their systematic review of evidence based indications of elbow arthroscopy supported the use of elbow arthroscopy in majority of conditions where it is currently used.

Elbow arthroscopy is a valuable tool for both diagnostic and therapeutic purpose. Minimal invasiveness and effective rehabilitation after the surgery helps the patient achieve an early recovery and facilitates return to normal activities of daily living [20]. However, elbow arthroscopy remains a technically difficult and challenging procedure with a higher potential for neurological complications hence it should be used judiciously by a vigilant surgeon with a fair knowledge of the arthroscopic anatomy. Moreover, in difficult situations, the surgeon should not hesitate to convert an arthroscopic procedure to an open procedure in order to facilitate a thorough treatment for any elbow pathology, especially in cases of infective pathology and severe adhesions.
